
JOURNAL INFORMATION
==============================
NLM Title Abbreviation: J Clin Hypertens (Greenwich)
Journal ID: J Clin Hypertens (Greenwich)
NLM Journal ID: 100888554
Journal ID: JCH

The Journal of Clinical Hypertension

ISSN: 1524-6175
EISSN: 1751-7176
Publisher: Wiley

ARTICLE INFORMATION
==============================
PMCID: PMC8029550
DOI: 10.1111/jch.13881
Article ID: JCH13881
Article version: 1
Subjects: Issue Information, Issue Information

Issue Information

Electronic publication date: 2021 Mar 26
Publication date: 2021 Mar
Volume: 23
Issue: 3
Issue title: Hypertension in Asia: From national guidelines to Asian consensus
First page: 399
Last page: 400
Copyright: © 2021 The Authors. The Journal of Clinical Hypertension published by Wiley Periodicals LLC.
License: This is an open access article under the terms of the http://creativecommons.org/licenses/by/4.0/ License, which permits use, distribution and reproduction in any medium, provided the original work is properly cited.
License URL: https://creativecommons.org/licenses/by/4.0/

Figure count: 0
Table count: 0
Page count: 3

source-schema-version-number: 2.0
cover-date: March 2021
details-of-publishers-convertor: Converter:WILEY_ML3GV2_TO_JATSPMC version:6.0.1 mode:remove_FC converted:06.04.2021

==============================
EDITOR-IN-CHIEF
Ji-Guang Wang

Volume 23 Issue 3 March 2021

### THE JCH THE JOURNAL OF CLINICAL HYPERTENSION

Open Access

**Special Issue:
Hypertension in Asia: From national
guidelines to Asian consensus**

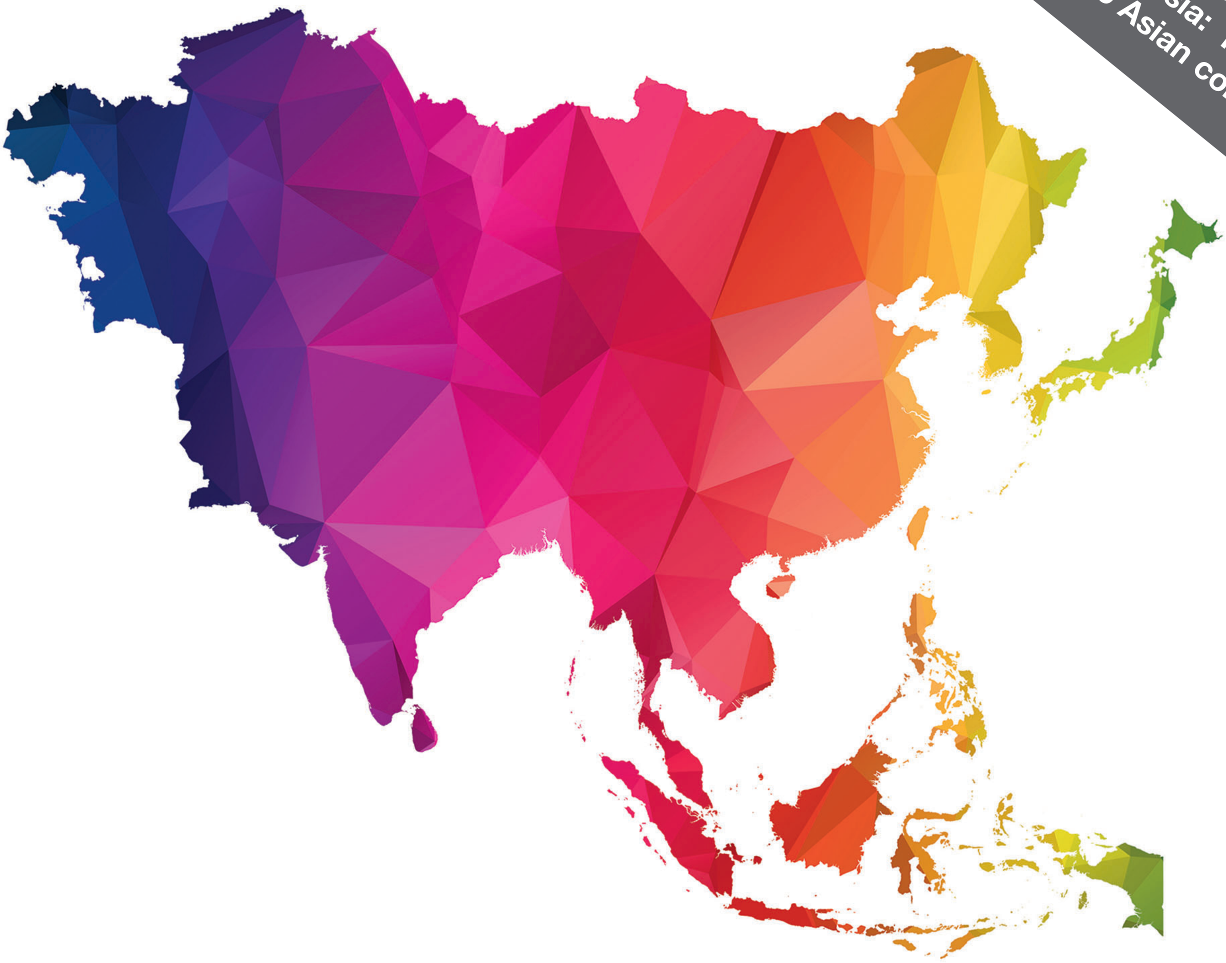

wileyonlinelibrary.com/journal/jch

WILEY

THE JCH The Journal of Clinical Hypertension®
HYPERTENSION
DIABETES
LIPIDS
OBESITY

Volume 23 | Number 3 | March 2021

### EDITOR IN CHIEF

**Ji-Guang Wang**
Shanghai Jiaotong University School of Medicine, Shanghai, China

### EDITOR IN CHIEF EMERITUS

**Michael A. Weber, MD**
New York, NY, USA

### ASSOCIATE EDITORS

**Chen-Huan Chen**
Taipei Veterens General Hospital, Taipei, Taiwan
**Julio Chirinos**
University of Pennsylvania, Philadelphia, PA, USA
**Ying-Qing Feng**
Guangdong General Hospital, Guangzhou, China
**Kazuomi Kario**
Jichi Medical University, Tochigi, Japan
**Yan Li**
Shanghai Jiaotong University School of Medicine, Shanghai, China
**Sungha Park**
Shinchonro Seodaemungu Yonsei Health System, Seoul, South Korea
**Alexandre Persu**
Universite Catholique de Louvain, Brussels, Belgium
**Yuging Zhang**
Fuwai Hospital, Beijing, China
**Zhiming Zhu**
Daping Hospital, Chongqing, China

### EDITORIAL BOARD

**Kei Asayama**
Teikyo University School of Medicine, Tokyo, Japan
**Jacques Blacher**
University Paris Descartes, Paris, France
**José Boggia**
Universidad de la República, Montevideo, Uruguay
**Louise M. Burrell**
The Univerity of Melbournce, Melbourne, Australia
**Xin Chen**
Ruijin Hospital North, Shanghai, China
**Hao-Min Cheng**
Taipei Veterens General Hospital, Taipei, Taiwan
**Yook-Chin Chia**
Sunway University, Bandar Sunway, Malaysia
**Peter W. de Leeuw**
Maastricht University, Maastricht, The Netherlands
**Takeshi Fujiwara**
Jichi Medical University School of Medicine, Shimotsuke, Japan
**Tibor Fulop**
Medical University of South Carolina, Charleston, SC, USA
**Peng Gao**
Daping Hospital, Chongqing, China
**Tine Willum Hansen**
Steno Diabetes Center Copenhagen, Gentofte, Denmark
**Satoshi Hoshide**
Jichi Medical University, Tochigi, Japan
**Jing Huang**
Chongqing Medical University, Chongqing, China
**Thomas Kahan**
Karolinska Institute, Stockholm, Sweden
**Reinhold Kreutz**
Charité Medical University, Berlin, Germany
**Li-Hua Li**
Dali University, Dali, Yunnan Province, China
**Ming Liu**
Jiangsu Province Hospital of Chinese Medicine, Nanjing, Jiangsu Province, China
**Pietro Amedeo Modesti**
University of Florence, Florence, Italy
**Masaki Mogi**
Ehime University, Matsuyama, Japan
**Jianjun Mu**
Xi’an Jiaotong University, Xi’an, China
**Teemu Niiranen**
University of Turku, Turku, Finland
**Akira Nishiyama**
Kagawa University, Takamatsu, Kagawa, Japan
**Augustine Odili**
University of Abuja, Abuja, Nigeria
**Takayoshi Ohkubo**
Teikyo University, Tokyo, Japan
**Georgianos Panagiotis**
Aristotle University of Thessaloniki, Athens, Greece
**Jeong-Bae Park**
Yonsei University Wonju College of Medicine, Wonju, Japan
**Giacomo Pucci**
University of Perugia, Perugia, Italy
**Markus P. Schlaich**
University of Western Australia, Perth, WA, Australia
**James Sharman**
University of Tasmania, Hobart, Australia
**Shigeru Shibata**
Teikyo University School of Medicine, Tokyo, Japan
**George Stergiou**
National and Kapodistrian University of Athens, Athens, Greece
**Koichi Tamura**
Yokohama City University, Yokohama, Japan
**Valerie Tikhonoff**
University of Padova, Padova, Italy
**Hirofumi Tomiyama**
Tokyo Medical University Tokyo Japan
**Kelvin Tsoi**
Chinese University of Hong Kong, Hong Kong
**Nashingh Verma**
King George's Medical University, Lucknow, India
**Tzung-Dau Wang**
National Taiwan University College of Medicine, Taipei, Taiwan
**Ting-Yan Xu**
The Shanghai Institute of Hypertension, Shanghai, China
**Hao Xue**
Chinese People's Liberation Army General Hospital, Beijing, China
**Jianzhong Xv**
Ruijin Hospital, Shanghai, China
**Yuichiro Yano**
Duke University, Durham, North Carolina, USA
**Tetsuro Yoshida**
Onga Nakama Medical Association Onga Hospital, Onga, Japan
**Jing Yu**
Lanzhou University, Lanzhou, Gansu Province, China

THE
### JCH
*The Journal of Clinical Hypertension*®

*The Journal of Clinical Hypertension*® (Online ISSN 1751–7176), is published in 12 issues per year.

Copyright © 2021 Wiley Periodicals LLC. All rights reserved. No part of this publication may be reproduced, stored or transmitted in any form or by any means without the prior permission in writing from the copyright holder. Authorization to copy items for internal and personal use is granted by the copyright holder for libraries and other users registered with their local Reproduction Rights Organisation (RRO), e.g. Copyright Clearance Center (CCC), 222 Rosewood Drive, Danvers, MA 01923, USA (www.copyright.com), provided the appropriate fee is paid directly to the RRO. This consent does not extend to other kinds of copying such as copying for general distribution, for advertising or promotional purposes, for creating new collective works or for resale. Permissions for such reuse can be obtained using the RightsLink "Request Permissions" link on Wiley Online Library. Special requests should be addressed to: permissions@wiley.com

**Disclaimer:** The Publisher and Editors cannot be held responsible for errors or any consequences arising from the use of information contained in this journal; the views and opinions expressed do not necessarily reflect those of the Publisher and Editors, neither does the publication of advertisements constitute any endorsement by the Publisher and Editors of the products advertised. *The Journal of Clinical Hypertension*® is not associated with any other hypertension specialty publication published in the USA or overseas.

View this journal online at wileyonlinelibrary.com/journal/JCH

The Journal of Clinical Hypertension accepts articles for Open Access publication. Please visit https://authorservices.wiley.com/author-resources/Journal-Authors/open-access/onlineopen.html for further information about OnlineOpen.

ISSN 1751-7176 (Online)

For submission instructions, subscription and all other information visit: wileyonlinelibrary.com/library/jch

**EDITORIAL**

**Managing Editor**
*Erica Byrd*
*jch@jjeditorial.com*

**Production Editor**
*Kavitha Raju*
*jch@wiley.com*

**PUBLISHING**

**Executive Editor**
*Christian Bjorbaek*
*cbjorbaek@wiley.com*

**Business Development Senior Manager**
*Chris Breslin*
*(443) 934-8925*
*cbreslin@wiley.com*

**Senior Advertising Account Executive** • *Karl Franz* • *(781) 388-8470* • *kfranz@wiley.com*

**Reprint Sales Director - Americas** • *Dave Surdel* • *(781) 388-8343* • *dsurdel@wiley.com*
